# Severe Craniofacial Trauma With Bilateral Epidural Hematomas and Complex Skull Base and Midface Fractures Managed With Early Multidisciplinary Damage Control Surgery and Immediate Osteosynthesis: A Case Report

**DOI:** 10.7759/cureus.106664

**Published:** 2026-04-08

**Authors:** Luis Alejandro Palacios Sánchez, Israel Raúl Hernández Galicia, Karla Fernanda Rosas Rosales, Natalia Jaqueline Barrera Pérez, María del Carmen Hernández Quesada

**Affiliations:** 1 Neurological Surgery/Spine Surgery, Hospital General Regional No. 2 “Dr. Guillermo Fajardo Ortiz”, Instituto Mexicano del Seguro Social (IMSS), Mexico City, MEX; 2 Oral and Maxillofacial Surgery, Hospital General Regional No. 2 “Dr. Guillermo Fajardo Ortiz”, Instituto Mexicano del Seguro Social (IMSS), Mexico City, MEX; 3 Medical Education, Hospital General Regional No. 2 “Dr. Guillermo Fajardo Ortiz”, Instituto Mexicano del Seguro Social (IMSS), Mexico City, MEX

**Keywords:** craniocerebral trauma, damage control surgery, epidural hematoma, facial fractures, internal fixation, le fort fractures, maxillofacial surgery, naso-orbito-ethmoid fractures, traumatic brain injury

## Abstract

Severe traumatic brain injury (TBI) associated with complex craniofacial fractures represents a life-threatening condition, requiring prompt, multidisciplinary management. Early intervention and timely access to surgical resources are critical factors influencing outcomes.

A 23-year-old male sustained a high-energy motorcycle accident without helmet use. On admission, the Glasgow Coma Scale (GCS) score was 13, followed by neurological deterioration. Cranial computed tomography (CT) demonstrated bilateral epidural hematomas, subdural hematoma, subarachnoid hemorrhage, and extensive craniofacial fractures, including Le Fort II and naso-orbito-ethmoidal (NOE) type III fractures, with skull base involvement. The patient underwent emergent, combined neurosurgical and maxillofacial damage control surgery, followed by early definitive reconstruction using osteosynthesis systems available in an emergency setting. Favorable clinical evolution was observed, achieving a GCS score of 15 at discharge without neurological deficits.

Early (<24 hours) multidisciplinary damage control surgery, combined with immediate availability of osteosynthesis materials, may significantly improve neurological and functional outcomes in patients with severe craniofacial trauma.

## Introduction

Traumatic brain injury (TBI) remains a leading cause of mortality and long-term disability worldwide, disproportionately affecting low- and middle-income countries (LMICs), where access to timely and specialized care is often limited [1-5]. High-energy mechanisms, particularly motorcycle-related accidents, frequently result in combined intracranial and complex craniofacial injuries, substantially increasing morbidity and mortality [6-10].

In Mexico, motorcycle-related trauma predominantly affects young male populations and is commonly associated with severe craniofacial and intracranial injuries [6]. These include skull fractures and intracranial hemorrhage [7,8], as well as complex maxillofacial fracture patterns, such as Le Fort and naso-orbito-ethmoidal (NOE) fractures [9,10].

The pathophysiology of TBI involves an initial primary insult, followed by a cascade of secondary injury mechanisms, including neuroinflammation, oxidative stress, and blood-brain barrier disruption. These processes result in increased vascular permeability, cerebral edema, and intracranial hypertension, with potential for rapid neurological deterioration within the first 24 hours [11,12]. This early period represents a critical therapeutic window for intervention to prevent irreversible brain injury and improve outcomes [11,12].

Computed tomography (CT) remains the gold standard for early evaluation, enabling rapid identification of lesions requiring urgent surgical management [13]. Early evacuation of intracranial hematomas has been consistently associated with improved survival [14-17].

Within this context, damage control surgery has emerged as a strategic approach focused on rapid physiological stabilization, particularly in settings where delays in definitive care may adversely impact outcomes [18-20]. However, its role in the integrated management of combined neurosurgical and maxillofacial trauma remains insufficiently characterized.

This case report aims to highlight the impact of early multidisciplinary damage control surgery, combined with immediate osteosynthesis, on clinical outcomes in severe craniofacial trauma, addressing a relevant gap in the current literature.

## Case presentation

A 23-year-old male, previously healthy, sustained a high-energy motorcycle collision without helmet use. Initial prehospital care was provided, followed by transfer to a private facility, where a delayed cranial CT was performed without surgical intervention. The patient was subsequently referred to our institution for definitive management.

Upon admission, vital signs were stable (blood pressure 146/85 mmHg, heart rate 70 bpm, respiratory rate 17 rpm), with a Glasgow Coma Scale (GCS) score of 13. The patient subsequently developed neurological deterioration, requiring endotracheal intubation and advanced airway management.

**Figure 1:**
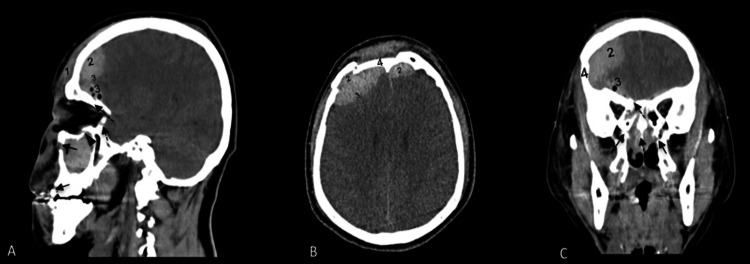
Initial non-contrast cranial and maxillofacial CT demonstrating severe traumatic brain injury associated with complex craniofacial fractures (A) Sagittal CT showing right frontoparietal subgaleal hematoma, with associated epidural and subdural hematomas in the setting of complex craniofacial fractures. (B) Axial CT demonstrating acute epidural and subdural hematomas along the right convexity, with a depressed right frontal bone fracture. (C) Coronal CT confirming a depressed frontal fracture with anterior cranial vault involvement. Maxillofacial CT shows a Le Fort II pattern, right orbital floor and medial wall fractures, and a type III NOE fracture. Annotations: (1) subgaleal hematoma; (2) epidural hematoma; (3) subdural hematoma; (4) depressed frontal fracture; arrows: Le Fort II components; arrowheads: orbital floor fracture; dashed arrows: NOE fracture. CT: computed tomography; NOE: naso-orbito-ethmoidal

**Figure 2:**
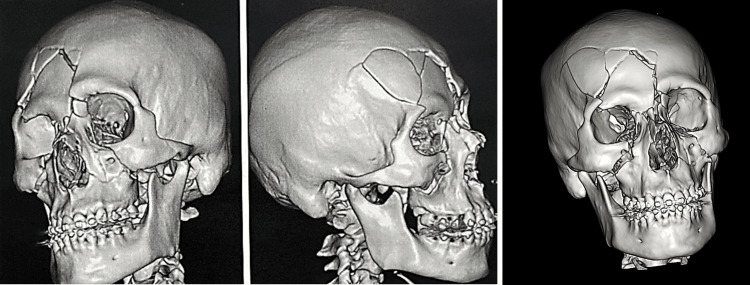
Three-dimensional CT reconstruction of the skull and facial skeleton Left and center: lateral views showing a multifragmented right frontoparietal fracture with depression and orbital involvement. Right: oblique view demonstrating extension to the orbit (roof, lateral wall, and floor), a Le Fort II fracture, and a right NOE type III injury. CT: computed tomography; NOE: naso-orbito-ethmoidal

Initial non-contrast cranial and facial CT demonstrated severe TBI, with bilateral epidural hematomas, subarachnoid hemorrhage, bifrontal contusions, and complex craniofacial fractures involving the frontal bone, orbital walls, nasal bones, maxilla, cribriform plate, and skull base (clivus), associated with pneumocephalus and hemosinus (Figures 1-2).

Given the patient’s neurological deterioration, the decision was made to proceed with emergent, combined neurosurgical and maxillofacial damage control surgery, based on radiological evidence of mass effect from epidural hematomas and the presence of complex craniofacial fractures involving the anterior skull base, necessitating urgent multidisciplinary intervention.

A bicoronal approach with right orbitozygomatic craniotomy was performed, allowing evacuation of epidural and subdural hematomas, frontal sinus cranialization, duraplasty with pericranium, and reconstruction of the anterior cranial fossa.

**Figure 3:**
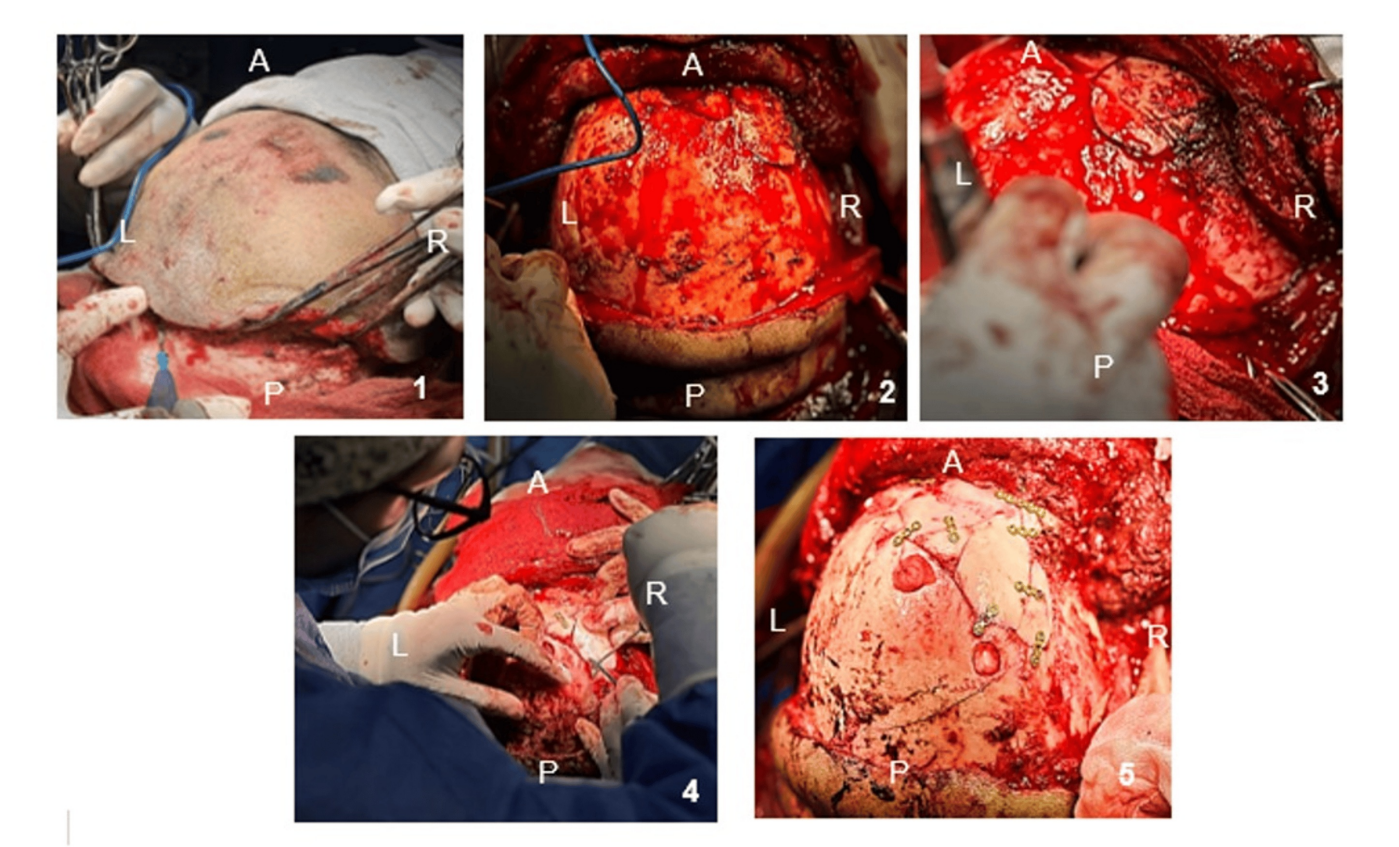
Intraoperative sequence via a bicoronal approach (1) Skin incision and initial flap dissection; (2) Surgical field exposure with adequate visualization of the anterior neurocranium; (3) Depressed right frontal fracture with exposure of the right temporalis muscle, dissected in a myocutaneous plane; (4) Reconstruction of the right anterior cranial fossa and orbital fixation using a miniplate and screw system; (5) Final result after right orbitozygomatic craniotomy and sequestrectomy, showing burr holes. Orientation: A = anterior, P = posterior, R = right, L = left.

Definitive craniofacial stabilization was achieved during the same procedure using a 2.0 mm miniplate osteosynthesis system, providing immediate structural stability (Figure 3).

The key surgical steps are demonstrated in Video 1, including the bicoronal approach, wide exposure of the anterior cranial vault, identification of comminuted frontal fractures, and definitive reconstruction using osteosynthesis fixation.

**Video 1:** Bicoronal approach and osteosynthesis reconstruction video Intraoperative video demonstrating the bicoronal approach and key steps of multidisciplinary damage control surgery, including surgical exposure, identification of frontal bone fractures, and osteosynthesis-based reconstruction.

Postoperative management included intensive care with neuroprotective measures, sedation, and hemodynamic support.

**Figure 4:**
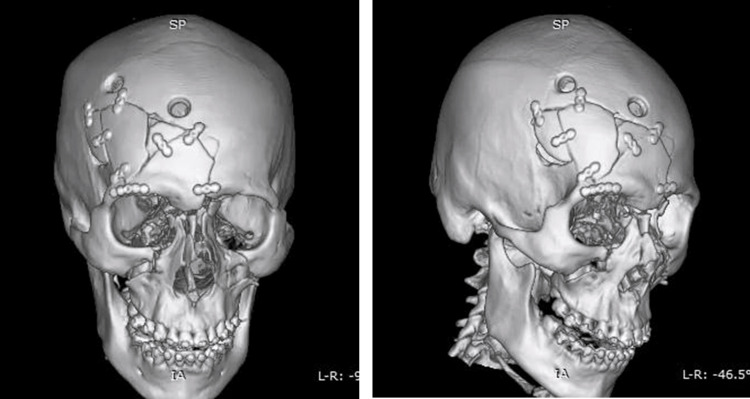
Postoperative three-dimensional CT reconstruction following the first surgical stage Left: frontal view; Right: oblique view. Findings demonstrate postsurgical changes after a bicoronal approach, right orbitozygomatic craniotomy, sequestrectomy, evacuation of epidural and subdural hematomas, frontal sinus cranialization with muscle interposition, duraplasty using pericranium, and reconstruction of the right anterior frontoparietal cranial fossa, with orbital fixation using miniplates and screws. CT: computed tomography

Follow-up imaging confirmed adequate evacuation of hematomas and reconstruction of craniofacial structures (Figure 4).

**Figure 5:**
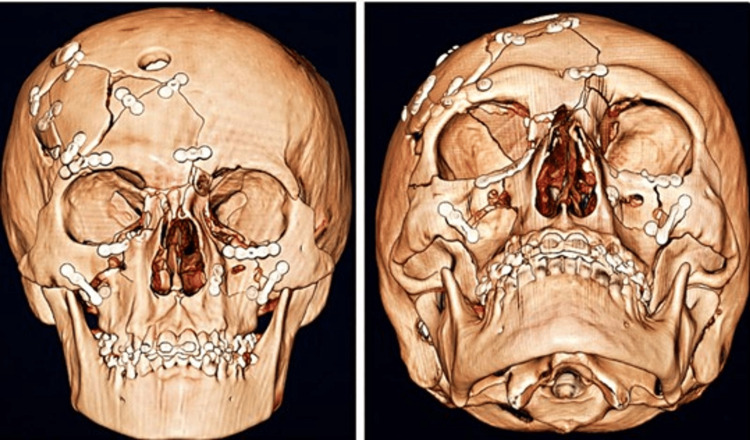
Postoperative three-dimensional CT reconstruction following the second surgical stage Left: frontal view; Right: basal view. Findings demonstrate postsurgical changes after open reduction and internal fixation via bilateral circumvestibular and bilateral subciliary approaches, including reconstruction of the facial buttresses, infraorbital rims, and the left nasomaxillary buttress. CT: computed tomography

A second-stage surgical procedure was performed for definitive open reduction and internal fixation (ORIF) of midface fractures, using circumvestibular and subciliary approaches, with restoration of facial buttresses and orbital rims (Figure 5).

The patient achieved complete neurological recovery, with a GCS score of 15 and no residual deficits.

## Discussion

This case highlights the clinical and epidemiological characteristics of severe motorcycle-related TBI, particularly in young male populations [6-8]. High-energy trauma mechanisms frequently result in multicompartmental intracranial hemorrhage and complex craniofacial fractures, which are associated with rapid neurological deterioration and increased mortality risk [7,8].

The presence of bilateral epidural hematomas is uncommon and represents a critical neurosurgical emergency. Early surgical evacuation is essential, as delays are associated with significantly worse outcomes [14,15]. The first 24 hours following injury constitute a crucial therapeutic window, during which timely intervention may prevent secondary brain injury and improve survival [11,12].

In addition to intracranial pathology, complex maxillofacial fractures, such as Le Fort II and NOE type III patterns, require coordinated multidisciplinary management [9,10,19,20]. These injuries reflect high-energy mechanisms and are frequently associated with skull base involvement, increasing both surgical complexity and morbidity [7].

Damage control surgery refers to a staged surgical strategy aimed at rapid physiological stabilization through hemorrhage control and temporary management of life-threatening injuries, followed by definitive reconstruction once the patient is stabilized [18-20]. In severe craniofacial trauma, this approach enables simultaneous management of intracranial pathology and structural facial instability, minimizing delays that could adversely affect neurological outcomes [14,15,20].

In the present case, the integration of neurosurgical and maxillofacial techniques within a single operative setting underscores the importance of multidisciplinary coordination. Early intervention facilitated not only timely hematoma evacuation but also immediate structural reconstruction, reducing the need for multiple staged procedures.

A critical factor contributing to the favorable outcome was the immediate availability of osteosynthesis systems, which enabled definitive fixation during the initial surgical intervention and avoided delays associated with staged reconstruction. This strategy may reduce operative time, hospital stay, and complication rates, particularly in resource-limited settings, where access to surgical materials is often delayed [18-20].

These findings support the concept that early, coordinated, and resource-optimized surgical strategies are essential to improving both neurological and functional outcomes in patients with complex craniofacial trauma [14,15,19,20].

## Conclusions

Severe craniofacial trauma associated with intracranial hemorrhage requires timely and coordinated multidisciplinary management, particularly within the first 24 hours following injury. This case suggests that early implementation of damage control principles, combined with the immediate availability of osteosynthesis systems, may improve both neurological and functional outcomes. These findings highlight the importance of institutional preparedness, timely decision-making, and integrated surgical strategies in the management of complex craniofacial injuries. Further studies are warranted to evaluate the reproducibility of this approach and to inform standardized management protocols, particularly in resource-limited settings.

## References

[1] Zhang Q, Li Y, Chang X (2023). Role of decompressive craniectomy in the management of traumatic brain injury - a meta-analysis of randomized controlled trials. Ann Indian Acad Neurol.

[2] Maas A, Menon D, Adelson P (2017). Traumatic brain injury: integrated approaches to improve prevention, clinical care, and research. Lancet Neurol.

[3] Ashraf M, Kamboh UA, Hussain SS (2022). Traumatic brain injury in underage motorcycle drivers: clinical outcomes and sociocultural attitudes from a lower-middle-income country. World Neurosurg.

[4] Faried A, Bachani AM, Sendjaja AN, Hung YW, Arifin MZ (2017). Characteristics of moderate and severe traumatic brain injury of motorcycle crashes in Bandung, Indonesia. World Neurosurg.

[5] Ojakapeli B, Nafula BJ, Seltzer L (2025). Patterns, severity, and outcomes of traumatic brain injury in a regional referral hospital in Kenya: a retrospective cohort study. Ann Med Surg (Lond).

[6] Castillo Cardiel MG, Flores Valdivia JL, González Ojeda A (2021). Facial fractures, surgical management, and outcomes in a tertiary hospital (Article in Spanish). Rev Esp Cir Oral Maxilofac.

[7] Alhathlol H, Alsikhan K, Alharbi T, Alsamaani I, Alhathloul A, Alyami R (2025). A 9 year retrospective review of motorcycle accidents at a level 1 trauma center in Riyadh. Surg Open Sci.

[8] Hwang IJ, Jeong TS, Kim WS, Kim JO, Jang MJ (2024). Epidemiology and outcomes of severe traumatic brain injury: regional trauma center in Incheon, Korea, 2018-2022. Korean J Neurotrauma.

[9] Ayyappan S (2024). Skull fracture patterns and morphologies among riders of fatal motorcycle accidents. Am J Forensic Med Pathol.

[10] Singaram M, Vijayabala GS, Udhayakumar RK (2016). Prevalence, pattern, etiology, and management of maxillofacial trauma in a developing country: a retrospective study. J Korean Assoc Oral Maxillofac Surg.

[11] Rajendra PB, Mathew TP, Agrawal A, Sabharawal G (2009). Characteristics of associated craniofacial trauma in patients with head injuries: an experience with 100 cases. J Emerg Trauma Shock.

[12] Carney N, Totten AM, O'Reilly C (2017). Guidelines for the management of severe traumatic brain injury, fourth edition. Neurosurgery.

[13] Maas AI, Marmarou A, Murray GD, Teasdale SG, Steyerberg EW (2007). Prognosis and clinical trial design in traumatic brain injury: the IMPACT study. J Neurotrauma.

[14] Bullock MR, Chesnut R, Ghajar J (2006). Surgical management of acute epidural hematomas. Neurosurgery.

[15] Seelig JM, Becker DP, Miller JD, Greenberg RP, Ward JD, Choi SC (1981). Traumatic acute subdural hematoma: major mortality reduction in comatose patients treated within four hours. N Engl J Med.

[16] Lizano Guevara F, Sáenz Araya D, Baizan Orias SD, Sevilla Torres E, Rojas Peláez A, Fernandez Vinocour D (2025). Facial fractures associated with craniomaxillofacial trauma in adults: a literature review. Cureus.

[17] Cooper DJ, Rosenfeld JV, Murray L (2011). Decompressive craniectomy in diffuse traumatic brain injury. N Engl J Med.

[18] Li A, Feng Q, Zhao Y, Zhang X, Jiang W (2025). Comprehensive meta-analysis of emergency trauma outcomes: trends, interventions, and survival rates. Front Public Health.

[19] Farber SJ, Nguyen DC, Skolnick GB, Woo AS, Patel KB (2016). Current management of zygomaticomaxillary complex fractures: a multidisciplinary survey and literature review. Craniomaxillofac Trauma Reconstr.

[20] Markowitz BL, Manson PN, Sargent L, Vander Kolk CA, Yaremchuk M, Glassman D, Crawley WA (1991). Management of the medial canthal tendon in nasoethmoid orbital fractures: the importance of the central fragment in classification and treatment. Plast Reconstr Surg.

